# The impact of early speech and language cognitive training care on the developmental quotient of children with language impairments: A retrospective study

**DOI:** 10.1097/MD.0000000000039898

**Published:** 2024-10-25

**Authors:** Yan Xue, Jian Xu, Dandan Liu

**Affiliations:** a Rehabilitation Department, Baoji People’s Hospital, Baoji, Shaanxi, China.

**Keywords:** clinical efficacy, developmental quotient, early speech and cognitive training, language disorders

## Abstract

This study aims to investigate the effects of early speech and language cognitive training on the nursing outcomes of children with language impairments and their impact on developmental quotient. From October 2018 to October 2023, the data of 80 children with language impairments treated at our hospital were selected. They were divided into an observation group (n = 40) and a control group (n = 40) based on the treatment plan. The control group received routine rehabilitation training, while the observation group received early cognitive language rehabilitation training. The treatment efficacy of the 2 groups, as well as differences in Gesell Developmental Schedule scores and serum indicators before and after treatment, were observed and analyzed. The treatment efficacy in the observation group was superior to that in the control group (*P* < .05), with a total effective rate of 92.50% in the observation group. After 6 months of treatment, the scores for adaptability, gross motor skills, fine motor skills, personal–social behavior, and language development quotient in the observation group were (77.41 ± 10.21), (77.15 ± 10.43), (80.43 ± 11.19), (71.14 ± 10.42), and (81.21 ± 12.03), respectively, significantly higher than those in the control group (*P* < .05). After 6 months of treatment, the mental development index and psychomotor development index in the observation group were (107.28 ± 10.43) and (96.60 ± 9.03), respectively, which were markedly higher than those in the control group (*P* < .05). After 6 months of treatment, the serum levels of insulin-like growth factor-1, 25-hydroxyvitamin D [25(OH)D3], and gamma-aminobutyric acid in the observation group were (52.43 ± 9.44) ng/mL, (31.45 ± 4.82) ng/mL, and (76.65 ± 10.54) µmol/mL, respectively. These levels were not significantly different from those in the control group (*P* > .05). The blood flow velocities of the middle cerebral artery, anterior cerebral artery, basal artery, and vertebral artery in the observation group and control group were compared after 6 months of treatment (*P* > .05). Early speech and cognitive training have shown promising efficacy in the treatment of language disorders, as it can improve developmental quotient in affected children and increase serum levels of insulin-like growth factor-1, 25(OH)D3, and gamma-aminobutyric acid.

## 1. Introduction

Children with language disorders typically exhibit significant deficits in both expressive and receptive language abilities compared to typically developing peers, which directly impacts their cognitive and social development, causing considerable distress to the affected children and their families.^[1,2]^ Early implementation of proactive intervention measures helps promote improvements in developmental quotient (DQ) for these children. However, effective pharmacological treatments have yet to be developed clinically, and the efficacy of solely language-based interventions remains limited.^[3]^ Speech and cognitive training is a rehabilitative approach that involves providing positive sensory, auditory, and visual stimuli in the early stages of pathology. This method aims to enhance cognitive behaviors such as thinking, memory, and perception in affected children. Currently, this technique is widely used in the treatment of conditions such as cerebral palsy and developmental delay, with some degree of success.^[4]^ However, there are few relevant studies on children with language impairment. We assume that children with language impairment can obtain better clinical manifestations and results through speech and cognitive training. Therefore, this study aims to explore the influence of early speech and cognitive training on the DQ of children with language impairment. The findings are reported below.

## 2. Materials and methods

### 2.1. General data

This retrospective study was approved by the Ethics Committee of Baoji People’s Hospital (2023-K01029). Eighty cases of children with language disorders treated in our hospital were selected within the time frame from October 2018 to October 2023. Inclusion criteria: (1) no history of intrauterine asphyxia. (2) Language barriers meet the “Zhu Futang practical pediatrics”^[5]^ in the standard. (3) Aged 2 to 6 years old. (4) Informed consent of the guardian of the child. Exclusion criteria: (1) language disorders caused by cleft palate, hearing impairment, etc. (2) Congenital heart disease, congenital developmental disorders and other congenital diseases. (3) Those with autism. (4) Combined with other serious diseases such as important organ diseases. According to the treatment plan, the patients were divided into an observation group (n = 85) and a control group (n = 45). The comparability of the 2 groups is shown in Table 1.

**Table 1:** Comparison of general data between observation group and control group.

Group	Number of cases	Gender		Age (years old)	Fetal age (week)
		Male	Female		
Observation group	40	21 (52.50)	19 (47.50)	4.05 ± 0.62	34.43 ± 1.10
Control group	40	22 (55.00)	18 (45.00)	4.01 ± 0.54	34.12 ± 1.09
*t*/*χ*^2^		0.050		0.308	1.266
*P*		.823		.759	.209

### 2.2. Treatment and evaluation methods

The control group received routine rehabilitation training. Approximately 1 hour after the child’s meal, when they were in good spirits, rehabilitation training was conducted. This training included activities such as lifting the head, rolling over, sitting, and grasping, with each session lasting for 15 minutes and conducted twice a day.

The observation group received early cognitive language rehabilitation training. Nursing staff guided parents to communicate with their children using gentle and affectionate tones. When the child cried, they used soothing phrases like “Oh, what’s wrong, sweetheart?” or “Good baby, mommy is here” to comfort them. If the child became agitated during training, the session was paused, and the child’s head and back were gently stroked for comfort. Small toys were also used to divert the child’s attention. When the child was in good spirits, nursing staff guided parents to engage in face-to-face activities with the child, such as opening the mouth, sticking out the tongue, and smiling, to improve their nonverbal communication skills. Patients whose movements were not accurate were patiently corrected. Simple and easy-to-read children’s songs and poems were recited to cultivate the child’s sense of language and reading habits. Pictures or toys were placed approximately 20 cm away from the child’s eyes for them to observe when awake. At the same time, nursing staff pointed to the pictures or toys and pronounced their names to promote the child’s cognitive development. Nursing staff used sound-producing toys like rattles and swung them at a distance of 10 cm from the child, observing the degree to which the child rotated their head along with the toy. This training lasted for 5 minutes per session and was conducted twice a day. Nursing staff instructed parents on the correct method of touching the child, providing tactile stimulation. They guided the child to grasp toys and touch the parent’s face, and rewarded the child with candy, small toys, or cheek kisses when the expected actions were completed. Nursing staff also guided parents to hold the child and rock them gently to promote the formation and development of the child’s sense of balance. Passive and active movements were used to promote the development of the child’s motor functions.

### 2.3. Check methods

Peripheral venous blood samples of 3 mL were collected from both groups of patients before treatment and after 6 months of treatment. The blood samples were allowed to stand at room temperature for half an hour before centrifugation at a speed of 3000 rpm for 10 minutes. Serum samples were obtained and analyzed using enzyme-linked immunosorbent assay to detect serum insulin-like growth factor-1 (IGF-1), 25-hydroxyvitamin D [25(OH)D3], and γ-aminobutyric acid (GABA). The reagent kits used were products of Shanghai Zenbio Biotechnology Co., Ltd., Shanghai, China, and the instrument used was a Siemens MK3 enzyme-linked immunosorbent assay analyzer.

Before treatment and after 6 months of treatment, transcranial Doppler ultrasonography was used to examine the cerebral arterial blood flow velocities, including middle cerebral artery (MDA), anterior cerebral artery (ACA), basal artery (BA), and vertebral artery (VA), in both groups of patients. The instrument used was the KJ-2V1M Transcranial Doppler Instrument manufactured by Nanjing Kejin Scientific Instrument Co., Ltd., Nanjing, China.

### 2.4. Criteria for evaluation

(1) The Gesell Developmental Schedule^[6]^ is utilized to assess the DQ of the children. This scale comprises 5 dimensions: gross motor, fine motor, adaptability, language, and personal–social behavior. Each dimension is scored from 0 to 100 points, with higher scores indicating better DQ in the children.
(2) The treatment effectiveness was evaluated using the Chinese Rehabilitation Center’s method for assessing language development delay.^[7]^ Complete recovery: the child’s recovery level reached stage 5 or above, and language function returned to normal. Significant improvement: the child’s language function improved by 2 stages compared to before treatment, with noticeable enhancement in language function. Effective: the child’s language function improved by 1 stage compared to before treatment, with some improvement in language function. Ineffective: there was no improvement in the child’s language function compared to before treatment.

### 2.5. Statistical processing

Data were analyzed using SPSS version 22.0 software. Measurement data, which include variables such as age, gestational age, and DQ, were presented as means ± standard deviations. Categorical data, including variables such as gender and treatment outcomes, were expressed as frequencies (n) and percentages (%). Differences between groups were assessed using appropriate statistical tests based on the data types. For comparisons involving continuous variables, the independent-samples *t* test was employed if the data followed a normal distribution; otherwise, the nonparametric Mann–Whitney *U* test was used. For categorical variables, the chi-square test (*χ*²) was applied to evaluate differences in proportions between groups. The significance level for all tests was set at α = 0.05. Statistical significance was determined by *P* < .05.

## 3. Results

### 3.1. Comparison of therapeutic effects between the 2 groups

The treatment effectiveness in the observation group was significantly better (Z = ‐3.572; *P* < .05), with a high total effective rate of 92.50%. See Table 2.

**Table 2:** Comparison of therapeutic effects between the 2 groups.

Group	Number of cases	Cured basically	Significantly effective	Effective	Ineffective
Observation group	40	5 (12.50)	18 (45.00)	14 (35.00)	3 (7.50)
Control group	40	2 (5.00)	11 (27.50)	16 (40.00)	11 (27.50)
Z		‐2.672			
*P*		.008			

### 3.2. The comparison of Gesell developmental scores before and after treatment in the 2 groups

The adaptability, gross motor skills, fine motor skills, personal–social behavior, and language development quotients significantly improved after treatment compared to before in both the observation and control groups (*P* < .05). After 6 months of treatment, the adaptability, gross motor skills, fine motor skills, personal–social behavior, and language development quotients were significantly higher in the observation group compared to the control group (*P* < .05). See Table 3.

**Table 3:** The comparison of Gesell developmental scores before and after treatment in the 2 groups.

Group	Number of cases	Adaptability		Gross motor		Fine motor	
		Before treatment	6 months after treatment	Before treatment	6 months after treatment	Before treatment	6 months after treatment
Observation group	40	52.21 ± 9.44	77.41 ± 10.21*	56.63 ± 9.17	77.15 ± 10.43*	52.21 ± 10.43	80.43 ± 11.19*
Control group	40	53.06 ± 9.10	68.89 ± 9.89*	57.12 ± 9.40	66.64 ± 11.18*	53.60 ± 11.18	71.13 ± 10.83*
Z		‐0.410	3.791	‐0.236	4.347	‐0.575	3.777
*P*		.683	.000	.814	.000	.567	.000

Group	Number of cases	Personal–social behavior			Language		
		Before treatment	6 months after treatment		Before treatment	6 months after treatment	
Observation group	40	51.56 ± 9.97	71.14 ± 10.42*		32.21 ± 9.44	81.21 ± 12.03*	
Control group	40	50.08 ± 9.82	63.34 ± 11.15*		31.80 ± 8.87	66.56 ± 11.43*	
*t*		0.669	3.233		0.200	5.584	
*P*		.506	.002		.842	.000	

* Compared with before treatment *P* < .05.

### 3.3. Comparison of MDI and PDI between the 2 groups before and after treatment

The mental development index (MDI) and psychomotor development index (PDI) scores increased significantly after treatment compared to before treatment in both the observation and control groups (*P* < .05). After 6 months of treatment, the MDI and PDI scores in the observation group were (107.28 ± 10.43) and (96.60 ± 9.03), respectively, which were significantly higher than those in the control group (*P* > .05). See Table 4.

**Table 4:** Comparison of MDI and PDI between the 2 groups before and after treatment.

Group	Number of cases	MDI		PDI	
		Before treatment	6 months after treatment	Before treatment	6 months after treatment
Observation group	40	81.12 ± 9.22	107.28 ± 10.43*	84.43 ± 8.82	96.60 ± 9.03*
Control group	40	82.28 ± 9.03	100.72 ± 12.03*	85.56 ± 8.12	90.12 ± 8.15*
*t*		‐0.568	2.606	‐0.596	3.369
*P*		.571	.011	.553	.001

* Compared with before treatment *P* < .05.

### 3.4. Comparison of serum indexes between the 2 groups before and after treatment

After treatment, the serum levels of IGF-1, 25(OH)D3, and GABA increased significantly compared to before treatment in both the observation and control groups (*P* < .05). After 6 months of treatment, the serum levels of IGF-1, 25(OH)D3, and GABA in the observation group were considerably higher than those in the control group (*P* < .05). See Table 5.

**Table 5:** Comparison of MDI and PDI between the 2 groups before and after treatment.

Group	Number of cases	MDI		PDI	
		Before treatment	6 months after treatment	Before treatment	6 months after treatment
Observation group	40	81.12 ± 9.22	107.28 ± 10.43*	84.43 ± 8.82	96.60 ± 9.03*
Control group	40	82.28 ± 9.03	100.72 ± 12.03*	85.56 ± 8.12	90.12 ± 8.15*
*t*		‐0.568	2.606	‐0.596	3.369
*P*		.571	.011	.553	.001

* Compared with before treatment *P* < .05.

### 3.5. Comparison of cerebral blood flow velocity before and after treatment in 2 groups

After 6 months of treatment, the blood flow velocities of MDA, ACA, BA, and VA were significantly increased compared to before treatment in both the observation and control groups (*P* < .05). The blood flow velocities of MDA, ACA, BA, and VA were compared between the observation group and the control group after 6 months of treatment (*P* > .05). See Table 6.

**Table 6:** Comparison of cerebral blood flow velocity before and after treatment in 2 groups.

Group	Number of cases	MDA (cm·s^‐1^)		ACA (cm·s^‐1^)		BA (cm·s^‐1^)		VA (cm·s^‐1^)	
		Before treatment	6 months after treatment	Before treatment	6 months after treatment	Before treatment	6 months after treatment	Before treatment	6 months after treatment
Observation group	40	45.60 ± 7.12	54.32 ± 7.65*	35.58 ± 5.80	45.54 ± 6.12*	31.83 ± 7.05	40.43 ± 5.67*	20.41 ± 5.43	25.51 ± 4.63*
Control group	40	45.43 ± 7.08	53.06 ± 7.80*	35.80 ± 5.73	44.83 ± 6.35*	30.95 ± 7.43	39.50 ± 6.07*	19.84 ± 5.50	25.14 ± 5.09*
t		0.107	0.729	-0.171	0.509	0.543	0.708	0.466	0.340
*P*		.915	.468	.865	.612	.588	.481	.642	.735

* Compared with before treatment *P* < .05.

## 4. Discussion

Language development is highly complex and adaptable, playing a crucial role in intellectual development. Children with language disorders often experience delayed language cognitive development.^[8–11]^ This is because the late stages of pregnancy are critical for fetal brain development, and children with fewer brain cells at birth may experience impacts on their physical and intellectual growth. Language-impaired children often have underdeveloped cerebral blood flow regulation and incomplete vascular development in the white matter areas of the brain, leading to a higher risk of cerebral hemorrhage and ischemic brain injury. Additionally, these children have a higher risk of developing respiratory distress syndrome, and rapid fluid expansion during neonatal resuscitation can lead to seizures, hypoglycemia, and hemorrhagic brain injury, which are also risk factors affecting their language and cognitive development.^[12,13]^

Early intervention for children with language disorders has been shown to improve their language and cognitive functions, thereby enhancing their overall development and quality of life.^[14]^ Speech and cognitive training, as a form of rehabilitation, have been found to promote growth and development in children when combined with early developmental training.^[15]^ In this study, it was observed that the treatment effectiveness in the observation group was superior to that in the control group. Furthermore, after 6 months of treatment, the observation group exhibited significantly higher scores in adaptability, gross motor skills, fine motor skills, personal–social behavior, and language development quotient compared to the control group. Additionally, both groups showed increased scores in MDI and PDI posttreatment compared to pretreatment scores, with the observation group showing significantly higher MDI and PDI scores compared to the control group. This result suggested that early speech and cognitive training have a positive therapeutic effect in the treatment of language disorders, improving the DQ of affected children and promoting intellectual and psychomotor development. This finding is consistent with previous clinical research conclusions. Early speech and cognitive training involve parents providing beneficial stimulation in various sensory modalities, such as perception, vision, and audition, which can enhance the child’s communicative attitude, improve perception, thinking, memory, and other cognitive behaviors, and improve the child’s tracking and gaze capabilities. This can help establish effective visual neural pathways, increase cortical excitability, and enhance cognitive functions, language comprehension, and expressive abilities in children with language disorders.^[16]^

IGF-1, or Insulin-like Growth Factor 1, is a growth-regulating hormone that plays a crucial role in growth, maintenance, and metabolic synthesis in infants and young children.^[17]^ 25(OH)D3 is an essential nutrient for infant bone development, which can influence their motor development.^[18]^ GABA, or gamma-aminobutyric acid, is an important neurotransmitter that can improve brain energy metabolism, promote acetylcholine synthesis and growth hormone secretion, and benefit the growth and development of children with language disorders.^[19]^ The study found that the levels of serum IGF-1, 25(OH)D3, and GABA in the observation group were significantly higher than those in the control group after 6 months of treatment. This suggests that early language cognitive training may elevate the serum levels of IGF-1, 25(OH)D3, and GABA in children with language disorders. This mechanism is crucial for enhancing the DQ of the children. During early language cognitive training, the use of gentle and affectionate tones by guiding parents in interacting with their children and providing correct touch can help calm their mental state and enhance their sense of security. Additionally, benign stimulation through facial expression interaction, reciting nursery rhymes and classical poetry, observing pictures and toys, and using rattles can assist in regulating the children’s neuroendocrine system, thereby increasing the secretion of hormones that promote growth and development.^[20]^

Children with language disorders may experience reduced cerebral blood flow due to cerebral hemorrhage or ischemic lesions, which can affect neuronal activity and synaptic transmission, thereby impacting the development of language and cognitive functions.^[21]^ This study found that the cerebral blood flow velocities of MDA, ACA, BA, and VA increased after 6 months of treatment in both the observation and control groups compared to before treatment. However, there was no statistically significant difference in cerebral blood flow velocities of MDA, ACA, BA, and VA between the 2 groups after 6 months of treatment. This suggests that over time, cerebral blood perfusion gradually improves in children with language disorders, and early language cognitive training does not appear to have a significant effect on the cerebral blood flow perfusion status of these children. This study still has some limitations. First, this study is a retrospective study with certain bias; second, the sample size of this study is small, but this does not affect the accuracy of this study; meanwhile, children have many uncertain factors, and more comprehensive and perfect evaluation criteria are still needed.

In summary, early language cognitive training demonstrates favorable efficacy in the treatment of language disorders. It can improve the DQ of affected children as well as increase serum levels of IGF-1, 25(OH)D3, and GABA.

## Author contributions

**Conceptualization:** Yan Xue, Jian Xu, Dandan Liu.

**Data curation:** Yan Xue, Jian Xu, Dandan Liu.

**Formal analysis:** Yan Xue, Jian Xu, Dandan Liu.

**Funding acquisition:** Yan Xue.

**Investigation:** Yan Xue, Dandan Liu.

**Methodology:** Yan Xue, Jian Xu, Dandan Liu.

**Supervision:** Dandan Liu.

**Validation:** Jian Xu.

**Writing – original draft:** Yan Xue, Dandan Liu.

**Writing – review & editing:** Yan Xue, Dandan Liu.
